# Frailty Assessment Tools Influence the Outcome Associations Among Patients With Diabetes: A Retrospective Cohort Study

**DOI:** 10.1016/j.jacasi.2025.02.014

**Published:** 2025-04-22

**Authors:** Jui Wang, Szu-Ying Lee, Chia-Ter Chao, Jenq-Wen Huang, Kuo-Liong Chien

**Affiliations:** a Institute of Epidemiology and Preventive Medicine, College of Public Health, National Taiwan University, Taipei, Taiwan; b Health Data Research Center, National Taiwan University, Taipei, Taiwan; c Division of Nephrology, Department of Internal Medicine, National Taiwan University Hospital Yunlin branch, Yunlin County, Taiwan; d Division of Nephrology, Department of Internal Medicine, National Taiwan University Hospital and National Taiwan University College of Medicine, Taipei, Taiwan; e Graduate Institute of Toxicology, National Taiwan University College of Medicine, Taipei, Taiwan; f Graduate Institute of Medical Education and Bioethics, National Taiwan University College of Medicine, Taipei, Taiwan; g Division of Nephrology, Department of Internal Medicine, Min Sheng General Hospital, Taoyuan City, Taiwan

**Keywords:** cardiovascular event(s), chronic kidney disease, diabetes mellitus, frail renal phenotype, frailty, outcomes

## Abstract

**Background:**

Frailty, characterized by aging-associated physiological reserve decline, leads to functional loss and adverse outcomes. Patients with diabetes mellitus (DM) have a high frailty risk. However, whether frailty assessment results derived from different tools diverge regarding their outcome correlations remains unclear.

**Objectives:**

The authors analyzed associations between different frailty assessment results and DM patients’ outcomes

**Methods:**

Between 2008 and 2016, adults (age >40 years) with type 2 DM were identified from the National Taiwan University Hospital Integrated Medical Database. The frailty assessment was performed using modified FRAIL scale and frailty index. Cox proportional hazard and Poisson regression analyses were used to determine the relationship between frailty and multiple outcomes after multivariate adjustment.

**Results:**

In total, 30,012 patients (mean 64.1 years, 45.4% women) with type 2 DM were included. The 2 frailty assessments were moderately positively correlated (r = 0.49; 95% CI: 0.48-0.49). After a median of 7.1 years (Q1-Q3: 3.9-10.4 years) of follow-up, FRAIL-identified mild and moderate-to-severe frailty did not correlate with a high mortality probability, but frailty index–identified severe and moderate frailty did. However, FRAIL-identified moderate-to-severe frailty correlated with a higher probability of all-cause hospitalization (incidence rate ratio [IRR]: 1.2; 95% CI: 1.09-1.32), intensive care unit admission (IRR: 4.19; 95% CI: 1.69-10.38), and cardiovascular hospitalization (IRR: 1.46; 95% CI: 1.28-1.66), whereas frailty index–identified mild, moderate, and severe frailty increased the probability of all-cause and cardiovascular hospitalizations only.

**Conclusions:**

We observed major discrepancies in outcome associations between FRAIL scale and frailty index among DM patients. Carefully selecting tools for measuring DM-associated frailty is important.

Geriatric syndromes (GS) characterize the degenerative phenotypes among older adults not readily attributable to disease entities, but predispose affected individuals to adverse outcomes.^1^ Frailty, as an overarching GS, increases the susceptibility to insults through homeostasis perturbation. Meta-analyses show that the prevalence of frailty and prefrailty is high among community-dwelling older adults (14%-46%).^2^ Frailty is also highly prevalent in individuals with diabetes mellitus (DM),^3^^,^^4^ as a systematic review suggests that the prevalence of frailty in patients with DM ranges between 9% and 21%.^5^ Recent studies have disclosed that Taiwan ranks third to fourth among 21 countries or jurisdictions with regard to age- and sex-standardized incidence of DM, higher than almost all other Asian countries.^6^ Moreover, DM accounts for the majority of cases (>50%) with end-stage kidney disease in Taiwan. Considering the public health concern resulting from diabetes, more should be explored regarding how to manage complications associated with DM in Taiwan, especially frailty, an underappreciated complication.

Multiple contributors to incident frailty exist in those with DM. For instance, comorbidities, hypoglycemia, and antidiabetic medications can influence frailty risk through various mechanisms.^7^, ^8^, ^9^ In addition, frailty potentially interferes with how we care for these patients, and individualized management strategies are recommended for frail individuals with DM.^10^ Therefore, the consensus has recommended that frailty assessment becomes a routine aspect of DM care.^5^ Despite the perceived importance, tools for these assessment needs remain underexplored. A literature summary identifies considerable heterogeneity in the settings and approaches of assessment.^5^ Consequently, how to assess frailty in patients with DM requires more evidence for decision making, particularly in the Asia Pacific region. Prior international consensus dictated that tools for frailty assessment in this population should be based on clinical contexts.^11^ During frailty screening in primary care, gait speed, timed-up-and-go test and electronic frailty index (eFI) serve the purpose well, whereas in specialists’ clinics, the Fried phenotype, FRAIL scale, and short physical performance battery would be good choices.^11^ However, the existing literature rarely compares differences in outcome correlation between tools used for DM patients in primary care and in specialists’ clinics in Asia. This is particularly concerning, because Asian countries mostly face population aging, accompanied by a rising prevalence of DM and frailty concurrently.

We thus hypothesized that the association between frailty and outcomes in Asian DM patients might differ according to the assessment tools and types of outcomes. Specifically, we chose 2 instruments, an eFI and the FRAIL scale, that were recommended for assessing frailty in different clinical contexts among patients with DM for result comparison. We selected multiple generic and DM-related outcomes for analyses to discern the exact clinical applicability of each instrument, using a retrospective cohort design.

## Methods

### Ethical statement

The protocol of the current study, as part of the parent study, was approved by the ethical review board of the National Taiwan University Hospital (No. 201708098RIND). This study was conducted according to the principles of the Declaration of Helsinki. Informed consent was deemed unnecessary by the review board because of participant data anonymization before data provision and the retrospective nature of the study.

### Data source and cohort design

**Figure 1:**
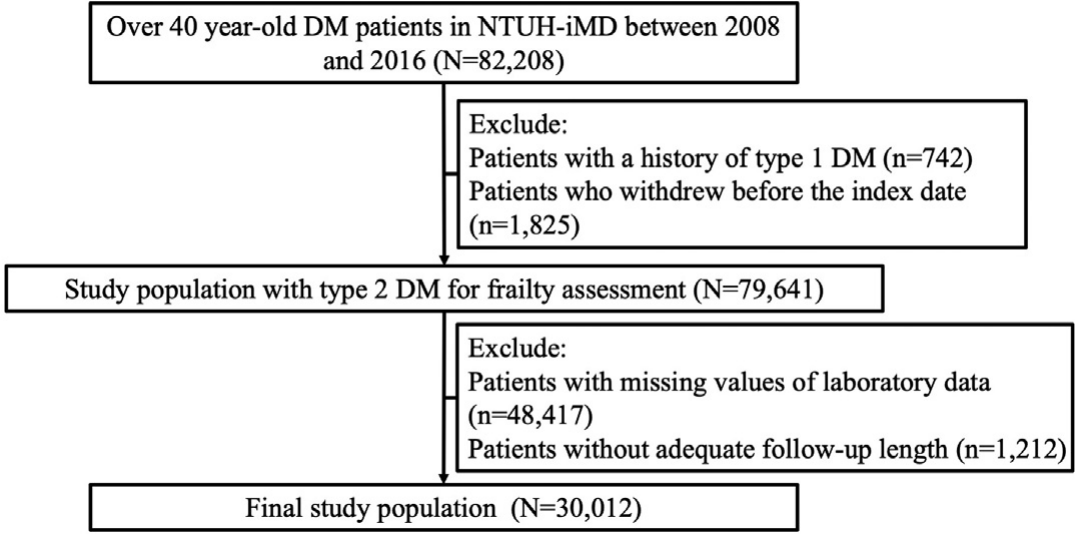
The Process of Study Participant Selection in the Current Study Adults older than age 40 years were retrospectively identified from our institute between 2008 and 2016. Following the application of exclusion criteria (screening phase), 96.9% were retained and entered into the selection phase. Among them, 30,012 (37.7%) were finally included for analyses. DM = diabetes mellitus; NTUH-iMD = National Taiwan University Hospital integrated medical database.

The clinical data was retrieved from the National Taiwan University Hospital Integrated Medical Database (NTUH-iMD). This data repository stores patient-level information continuously from the NTUH and its affiliated branches, which jointly provide care for citizens around Taiwan. Adults (age >40 years) with DM whose diagnoses were made by physicians, or having glycated hemoglobin levels over 6.5%, or fasting glucose levels >126 mg/dL, were retrospectively identified from the NTUH-iMD between 2008 and 2016 (Figure 1). We excluded patients with a diagnosis of type 1 DM and those who withdrew from providing data for the NTUH-iMD before the index date, defined as the date when the DM diagnosis was made. Those with missing laboratory values or <6 months of follow-up were also excluded; the remaining individuals constituted the type 2 DM analytic cohort. We recorded demographic profiles, body mass index (BMI), comorbidities, medications (including blood pressure-lowering, antidiabetic, antilipidemic, antiplatelet, anticoagulant, and anti-inflammatory), and laboratory data at baseline. We also assessed the frailty status using the methods outlined below. Patients were followed up until December 31, 2016, or until mortality, whichever occurred first.

### Frailty assessment approaches

The primary exposure variable was frailty status, which was determined using 2 approaches. First, the FRAIL scale was used to classify the presence and severity of frailty in the study participants. The FRAIL scale was introduced over a decade ago to screen for frailty among older adults.^12^ This scale comprises 5 components: fatigue, resistance, ambulation, illness, and weight loss. The FRAIL scale results can predict functional status, the risk of hospitalization and mortality among older adults and patients with DM, and other morbidities.^13^ Patients without any positive component are considered robust. Patients with more FRAIL components have increasing frailty severity with poorer outcomes.^12^^,^^13^ We previously operationalized the FRAIL scale based on prespecified diagnosis clustering, physical conditions, and performance in different local cohorts. The diagnostic clustering and other clinical features utilized to screen for each FRAIL component were selected based on keyword search within electronic medical records accompanied by an in-depth literature review for suitability, followed by review from local experts in geriatrics and epidemiology. The results showed significant associations with nutritional parameters, organ dysfunction, cognitive impairment, and worsening outcomes in patients with metabolic dysfunction and kidney diseases.^14^, ^15^, ^16^

Second, we used the index approach to assess frailty, based on the deficit cumulation model, to account for frailty’s biological origin.^17^ This model considers 38 types of health deficits and assigns 1 score to each if positive. Patients are given a summary deficit count score and then categorized into quartiles, with the higher quartiles suggesting greater frail severities. This model has been validated in a nationwide cohort with excellent outcome predictive efficacy and potentially assists in risk stratification among older adults and those with morbidities.^17^^,^^18^ We further pretested the applicability of this multimorbidity frailty index (FI) in our cohort of patients with DM, based on recommendations outlined recently.^19^ None of the 38 health deficits had missing values in our data set, and we recorded these deficits dichotomously. The prevalence of these 38 deficits in our patients were all lower than 80% (max, 62.43%). In addition, these health deficits correlated positively with increasing age, whereas none of the correlation coefficient between any of these 38 deficits exceeded 0.2.

### Outcome determination

The primary outcome of this study was the overall survival during follow-up, which was verified in the NTUH-iMD through data linkage. Secondary outcomes included all-cause hospitalization, cardiovascular disease (CVD)–related hospitalization, and intensive care unit (ICU) admission during the entire follow-up period, indicating that patients might have experienced more than one episode. The causes of hospitalization were determined based on the primary diagnoses recorded at discharge, and CVD included myocardial infarction, ischemic heart disease, heart failure, myocardial infarction, and cerebrovascular events.

### Statistical analyses

Continuous variables were described as mean ± SD or medians (Q1-Q3) for those with normal or skewed distribution, respectively. Categorical variables were expressed as numbers and percentages. Continuous and categorical variables were compared between groups using 1-way analysis of variance (>2 groups) and chi-square tests, respectively. We first evaluated the correlation between the FRAIL scale and the FI results in these patients using Pearson’s correlation. At baseline, clinical features and frailty assessment results were compared between patients with type 2 DM and those with or without different severities of frailty using the FRAIL scale or FI. After follow-up, we performed Kaplan-Meier analyses to examine the association between frailty severity and overall survival using log-rank tests for comparisons. Cox proportional hazard regressions were later used to estimate the mortality probability associated with different frailty assessment results (FRAIL scale or FI), accounting for all clinical features. The proportional hazards assumption for the Cox models was evaluated using graphical methods, and a global test based on Schoenfeld residuals. For the probability estimation of all-cause hospitalization, CVD-related hospitalization, and ICU admission, we calculated the incidence rate by the number of events during the follow-up period and estimated the incidence rate ratios (IRRs) for outcomes using Poisson regression models, incorporating the same set of variables. A prespecified sensitivity analysis included only patients receiving antidiabetic drugs to detect the influence of treatment intensity. We also performed a subgroup analysis based on patients’ age strata (≥65 years vs <65 years) to evaluate the applicability of the results in older and younger adults. In all analyses, a *P* value <0.05 was considered statistically significant.

## Results

A total of 82,208 adult patients with DM were identified from the NTUH-iMD during the study period (Figure 1). After applying the exclusion criteria, 30,012 patients with type 2 DM were included. The mean age of these patients was 64.1 ± 11.0 years, 45.4% (13,626 of 30,012) were women, and the median BMI was 25.0 kg/m^2^ (Table 1). The most common comorbidity was hypertension (64.2%, 19,266 of 30,012), followed by hyperlipidemia (49.0%, 14,714 of 30,012) and acute coronary syndrome (27.5%, 8,253 of 30,012). Less than one-fifth of the study patients had other organ illnesses, including kidney, liver, and pulmonary diseases.

**Table 1:** Clinical Features of Study Cohort According to the FRAIL Scale Results

	Total (N = 30,012)	FRAIL Scale Results			*P* Value
		Robust (Score 0) (n = 25,061)	Mild (Score 1) (n = 4,659)	Moderate to Severe (Score≧2) (n = 292)	
Demographic and physical data					
Age, y	64.1 ± 11.0	63.3 ± 10.7	68.3 ± 11.2	70.5 ± 12.3	<0.01
Female	13,626 (45.4)	11,352 (45.3)	2,125 (45.6)	149 (51.0)	0.14
BMI, kg/m^2^	25.0 (22.7-27.7)	25.0 (22.7-27.7)	24.8 (22.4-27.7)	24.1 (21.5-26.7)	<0.01
Comorbidity profile					
Hypertension	19,266 (64.2)	14,987 (59.8)	4,041 (86.7)	238 (81.5)	<0.01
Hyperlipidemia	14,714 (49.0)	12,082 (48.2)	2,474 (53.1)	158 (54.1)	<0.01
Atrial fibrillation	2,735 (9.1)	1,935 (7.7)	735 (15.8)	65 (22.3)	<0.01
Acute coronary syndrome	8,253 (27.5)	6,333 (25.3)	1,804 (38.7)	116 (39.7)	<0.01
Congestive heart failure	2,057 (6.9)	823 (3.3)	1,151 (24.7)	83 (28.4)	<0.01
Cerebrovascular disease	1,615 (5.4)	919 (3.7)	655 (14.1)	41 (14.0)	<0.01
Prior myocardial infarction	1,079 (3.6)	523 (2.1)	531 (11.4)	25 (8.6)	<0.01
Peripheral vascular disease	828 (2.8)	590 (2.4)	218 (4.7)	20 (6.9)	<0.01
Chronic kidney disease	4,286 (14.3)	2,135 (8.5)	2,024 (43.4)	127 (43.5)	<0.01
Cancer	3,653 (12.2)	2,318 (9.3)	1,251 (26.9)	84 (28.8)	<0.01
Chronic liver disease	3,814 (12.7)	3,132 (12.5)	623 (13.4)	59 (20.2)	<0.01
COPD	540 (1.8)	193 (0.8)	313 (6.7)	34 (11.6)	<0.01
Medication usage					
Antihypertensives					
ACEI	2,232 (7.4)	1,852 (7.4)	364 (7.8)	16 (5.5)	0.26
ARB	12,598 (42.0)	10,155 (40.5)	2,321 (49.8)	122 (41.8)	<0.01
β-blockers	7,279 (24.3)	5,929 (23.7)	1,286 (27.6)	64 (21.9)	<0.01
CCB	10,269 (34.2)	8,102 (32.3)	2,068 (44.4)	99 (33.9)	<0.01
Diuretic agents	6,983 (23.3)	5,168 (20.6)	1,709 (36.7)	106 (36.3)	<0.01
α-blockers	2,513 (8.4)	1,836 (7.3)	648 (13.9)	29 (9.9)	<0.01
Antilipidemics	13,620 (45.4)	11,325 (45.2)	2,178 (46.8)	117 (40.1)	0.03
Antiplatelets	11,172 (37.2)	8,739 (34.9)	2,311 (49.6)	122 (41.8)	<0.01
Anticoagulants	1,059 (3.5)	748 (3.0)	289 (6.2)	22 (7.5)	<0.01
Nonsteroidal anti-inflammatory drugs	3,541 (11.8)	2,504 (10.0)	964 (20.7)	73 (25.0)	<0.01
Antidiabetic drugs					<0.01
OAD monotherapy	7,002 (23.3)	5,778 (23.1)	1,161 (24.9)	63 (21.6)	
OAD combination therapy	11,893 (39.6)	10,366 (41.4)	1,450 (31.1)	77 (26.4)	
Insulin	2,072 (6.9)	1,599 (6.4)	454 (9.7)	19 (6.5)	
Laboratory data					
Fasting glucose, mg/dL	132.1 ± 47.9	133.2 ± 46.6	126.8 ± 53.9	122.2 ± 42.7	<0.01
Glycated hemoglobin,	7.06 ± 1.40	7.11 ± 1.41	6.84 ± 1.34	6.51 ± 1.11	<0.01
Total cholesterol, mg/dL	182.3 ± 41.4	183.3 ± 41.3	177.6 ± 42.0	171.4 ± 39.9	<0.01
Triglyceride, mg/dL	158.3 ± 121.2	158.5 ± 122.7	158.3 ± 114.5	140.5 ± 93.0	0.04
HDL cholesterol, mg/dL	43.2 ± 11.6	43.3 ± 11.4	42.9 ± 12.4	44.1 ± 12.9	0.03
LDL cholesterol, mg/dL	103.9 ± 34.2	104.8 ± 33.8	99.7 ± 35.9	99.5 ± 33.6	<0.01
Creatinine, mg/dL	1.2 ± 1.2	1.1 ± 1.0	1.6 ± 1.9	1.4 ± 1.5	<0.01
eGFR, mL/min/1.73 m^2^	73.2 ± 25.9	74.8 ± 24.4	64.2 ± 30.8	72.8 ± 32.0	<0.01
AST, U/L	29.1 ± 56.1	29.2 ± 59.0	28.8 ± 39.2	28.2 ± 21.7	0.87
ALT, U/L	29.6 ± 31.2	30.2 ± 30.2	27.0 ± 36.6	22.7 ± 17.5	<0.01
Frailty assessment results					
FRAIL scale	0.0 (0.0-0.0)	0.0 (0.0-0.0)	1.0 (1.0-1.0)	2.0 (2.0-2.0)	<0.01
Frail index	0.05 (0.03-0.11)	0.05 (0.03-0.08)	0.13 (0.11-0.18)	0.18 (0.13-0.26)	<0.01
Median follow-up duration,^a^ y	7.1 (3.9-10.4)	7.6 (4.2-10.7)	5.0 (2.7-7.8)	3.5 (1.5-5.5)	
Median follow-up duration,^b^ y	5.9 (3.0-9.2)	6.4 (3.4-9.6)	3.9 (2.0-6.5)	2.9 (1.2-4.7)	

Values are mean ± SD, n (%), or median (Q1-Q3).

ACEI = angiotensin-converting enzyme inhibitor; ALT = alanine transaminase; ARB = angiotensin receptor blocker; AST = aspartate transaminase; BMI = body mass index; CCB = calcium channel blocker; COPD = chronic obstructive pulmonary disease; eGFR = estimated glomerular filtration rate; HDL = high-density lipoprotein; LDL = low density lipoprotein; OAD = oral antidiabetic drug.

a For mortality.

b For other outcomes.

Using the FRAIL scale, these patients were divided into robust (score 0 [83.5%, 25,061 of 30,012]), mild frailty (score 1 [15.5%, 4,659 of 30,012]), and moderate-to-severe frailty (score ≥2 [0.97%, 292 of 30,012]) groups (Table 1). Patients with FRAIL-identified moderate-to-severe frailty were significantly older (*P <* 0.01) and had a higher prevalence of most comorbidities (*P <* 0.01) but had lower BMI (*P <* 0.01) than those in the mild frailty and robust groups. Patients with FRAIL-identified mild and moderate-to-severe frailty were more likely to take most medications (*P <* 0.01), except for angiotensin-converting enzyme inhibitors (*P =* 0.26) and antilipidemics (*P =* 0.03) (Table 1) than those in the robust group. Patients with mild frailty had the highest prevalence of oral antidiabetic monotherapy and insulin use, whereas those who were robust were more likely to receive oral antidiabetic combination therapy (*P <* 0.01). Regarding laboratory data, FRAIL-identified moderate-to-severe frailty was associated with significantly lower glycemic and total cholesterol levels but higher serum creatinine levels than the robust group (Table 1).

Using the FI approach, we also divided these patients based on the quartiles of FI results according to prior studies,^17^ yielding 4 groups with increasing frailty severity (fit, 30.2% [9,068 of 30,012]; mild, 20.4% [6,128 of 30,012]; moderate, 27.6% [8,282 of 30,012]; and severe, 21.8% [6,534 of 30,012]) (Supplemental Table 1). Patients with FI-identified severe frailty were significantly older (*P <* 0.01) and had a higher prevalence of all comorbidities (*P <* 0.01) than those with moderate or mild frailty and those who were fit. Patients with FI-identified severe frailty were more likely to take most medications (*P <* 0.01), except for angiotensin-converting enzyme inhibitors (Supplemental Table 1) than those with moderate or mild frailty and those who were fit. Regarding laboratory data, FI-identified severe frailty was associated with significantly lower glycemic and total cholesterol levels but higher serum creatinine levels than the other 3 groups (Supplemental Table 1).

**Figure 2:**
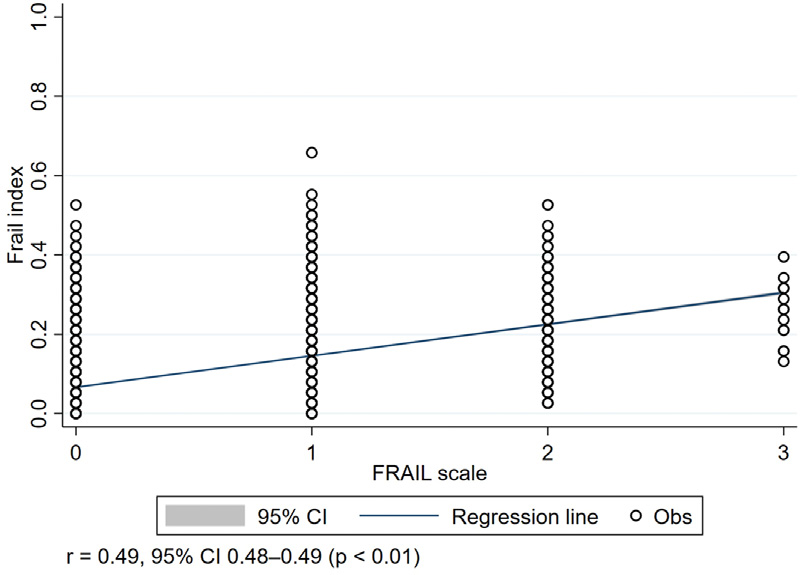
Correlation Plot Between FRAIL Scale and Frail Index Results Among included patients with type 2 diabetes, FRAIL scale results and frail index results exhibited moderate correlation (*r* = 0.49; 95% CI: 0.48-0.49; *P* < 0.01). obs = observation.

Next, we evaluated the relationship between the FRAIL scale and FI results. Patients with FRAIL-identified moderate-to-severe frailty also had a significantly higher FI than those with mild frailty and robust individuals (severe vs mild vs robust, 0.18 vs 0.13 vs 0.05; *P* < 0.01) (Table 1). Patients with FI-identified increasing frailty severity also had rising FRAIL scales (severe vs moderate vs mild vs fit, 0.50 ± 0.57 vs 0.16 ± 0.39 vs 0.06 ± 0.25 vs 0.03 ± 0.17; *P* < 0.01) (Supplemental Table 1). The 2 frailty assessments were moderately positively correlated in patients with type 2 DM (r = 0.49; 95% CI: 0.48-0.48; *P* < 0.01) (Figure 2).

**Figure 3:**
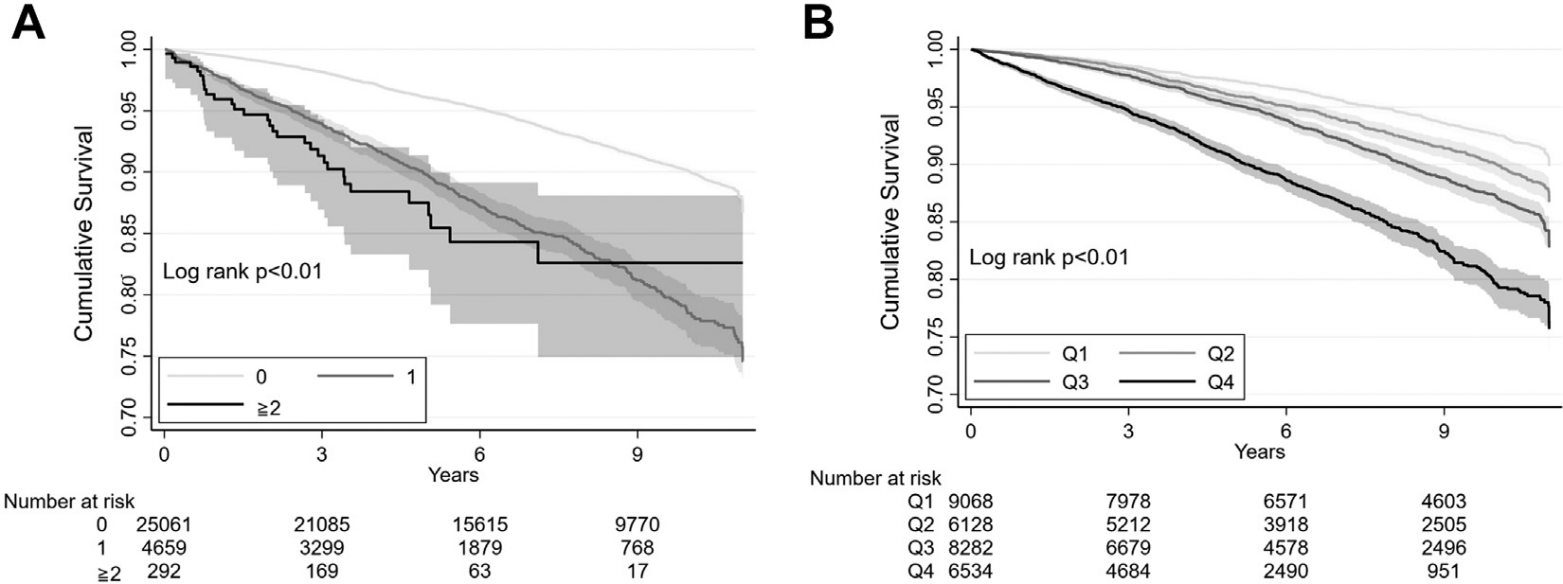
Kaplan-Meier Curves According to FRAIL and Index Categories (A) Using the FRAIL scale, patients with type 2 diabetes and increasing frailty severity (scores 0, 1, and ≥2) had progressively lower survival over follow-up (*P <* 0.01). (B) Using the frail index, those with type 2 diabetes and increasing frailty severity (quartiles 1, 2, 3, and 4) had progressively lower survival over follow-up (*P <* 0.01).

After a median of 7.1 years (Q1-Q3: 3.9-10.4 years) of follow-up, 8.0% (2,415 of 30,012) patients died. Patients with FRAIL-identified moderate-to-severe and mild frailty had significantly higher mortality rates than those who were robust (*P <* 0.01) (Figure 3A). Similarly, patients with a higher FI-identified severity had significantly increased mortality over time (*P <* 0.01) (Figure 3B). Having FRAIL-identified moderate-to-severe frailty significantly increased the probability of mortality by approximately 3-fold (HR: 3.18; 95% CI: 2.23-4.54), followed by FRAIL-identified mild frailty (HR: 2.46; 95% CI: 2.24-2.71) (Table 2). FI-identified severe frailty also increased the probability of mortality (HR: 3.09; 95% CI: 2.76-3.45), followed by moderate (HR: 1.77; 95% CI: 1.58-1.97) and mild frailty (HR: 1.36; 95% CI: 1.21-1.54) (Table 2).

**Table 2:** Risk of Primary Outcome Associated With 2 Frailty Assessment Results

	Number of Events	Total Population	Person-Years	Incidence Density^a^	Crude		Model 1^b^		Model 2^c^	
					HR	95% CI	HR	95% CI	HR	95% CI
Overall population										
Mortality										
FRAIL scale										
0	1,816	25,061	178,657.2	10.16	1.00		1.00		1.00	
1	568	4,659	24,755.8	22.94	2.46	2.24-2.71^d^	0.98	0.86-1.12	1.02	0.90-1.16
≧2	31	292	1,139.7	27.20	3.18	2.23-4.54^d^	1.05	0.72-1.52	1.20	0.83-1.74
Per 1 score					2.23	2.06-2.42^d^	0.99	0.88-1.11	1.03	0.92-1.16
Frail index										
Fit	556	9,068	71,919.0	7.73	1.00		1.00		1.00	
Mild	459	6,128	44,587.2	10.29	1.36	1.21-1.54^d^	1.13	0.99-1.29	1.14	1.00-1.30
Moderate	692	8,282	53,941.0	12.83	1.77	1.58-1.97^d^	1.20	1.05-1.36^d^	1.18	1.04-1.35^e^
Severe	708	6,534	34,105.5	20.76	3.09	2.76-3.45^d^	1.43	1.23-1.66^d^	1.44	1.23-1.67^d^
Per 1 quartile					1.44	1.39-1.50^d^	1.12	1.07-1.17^d^	1.12	1.06-1.17^d^
Age ≥65 y										
Mortality										
FRAIL scale										
0	1,191	10,814	77,879.8	15.29	1.00		1.00		1.00	
1	410	2,827	14,803.8	27.70	1.97	1.76-2.21^d^	0.93	0.80-1.09	0.96	0.82-1.12
≧2	23	187	689.0	33.38	2.61	1.73-3.95^d^	0.97	0.63-1.50	1.11	0.72-1.71
Per 1 score					1.85	1.68-2.05^d^	0.94	0.82-1.08	0.98	0.85-1.12
Frail index										
Fit	274	2,676	21,813.5	12.56	1.00		1.00		1.00	
Mild	303	2,564	19,544.4	15.50	1.25	1.06-1.48^d^	1.07	0.91-1.27	1.09	0.92-1.29
Moderate	494	4,267	29,112.1	16.97	1.41	1.22-1.64^d^	1.07	0.91-1.26	1.07	0.91-1.27
Severe	553	4,321	22,902.7	24.15	2.18	1.88-2.52^d^	1.18	0.98-1.42	1.20	1.00-1.45^e^
Per 1 quartile					1.29	1.23-1.35^d^	1.05	0.99-1.11	1.06	0.99-1.12
Age <65 y										
Mortality										
FRAIL scale										
0	625	14,247	100,777.4	6.20	1.00		1.00		1.00	
1	158	1,832	9,952.0	15.88	2.82	2.36-3.36^d^	1.18	0.94-1.49	1.25	0.99-1.58
≧2	8	105	450.7	17.75	3.43	1.70-6.89^d^	1.20	0.58-2.46	1.31	0.63-2.71
Per 1 score					2.50	2.15-2.91^d^	1.15	0.94-1.42	1.22	0.99-1.50
Frail index										
Fit	282	6,392	50,105.5	5.63	1.00		1.00		1.00	
Mild	156	3,564	25,042.9	6.23	1.15	0.94-1.40	1.21	0.98-1.49	1.22	099-1.50
Moderate	198	4,015	24,828.9	7.97	1.55	1.30-1.87^d^	1.42	1.15-1.77^d^	1.41	1.14-1.76^d^
Severe	155	2,213	11,202.8	13.84	2.90	2.38-3.54^d^	1.99	1.52-2.59^d^	1.99	1.52-2.61^d^
Per 1 quartile					1.38	1.30-1.48^d^	1.24	1.14-1.35^d^	1.24	1.14-1.35^d^

a Per 1,000 patient-years.

b Incorporating demographic and physical data, comorbidities, and medications.

c Incorporating model 1 variable and laboratory data.

d *P <* 0.01.

e *P <* 0.05.

Interestingly, after multivariate adjustment incorporating clinical features, FRAIL-identified mild (HR: 1.02; 95% CI: 0.90-1.16) and moderate-to-severe frailty (HR: 1.20; 95% CI: 0.83-1.74) were not associated with an increased probability of mortality among patients with type 2 DM. Conversely, FI-identified severe (HR: 1.44; 95% CI: 1.23-1.67) and moderate frailty (HR: 1.18; 95% 95% CI: 1.04-1.35) remained significantly associated with a higher mortality probability compared with the fit group (Table 2). For every increase in one FI-identified category, there was a 12% increase in mortality probability (HR: 1.12; 95% CI: 1.06-1.17).

We further divided participants into older (46.1%, 13,828 of 30,012) and younger groups (53.9%, 16,184 of 30,012). Among older adults, having FRAIL-identified moderate-to-severe frailty significantly increased the probability of mortality (HR: 2.61; 95% CI: 1.73-3.95), followed by FRAIL-identified mild frailty (HR: 1.97; 95% CI: 1.76-2.21) (Table 2). FI-identified severe frailty in older adults also increased the probability of mortality (HR: 2.18; 95% CI: 1.88-2.52), followed by moderate (HR: 1.41; 95% CI: 1.22-1.64) and mild frailty (HR: 1.25; 95% CI: 1.06-1.48) (Table 2). However, after multivariate adjustment, FRAIL-identified mild and moderate-to-severe frailty remained insignificantly associated with mortality probability. Conversely, FI-identified severe frailty (HR: 1.20; 95% CI: 1.00-1.45) was significantly associated with a higher mortality probability compared with the fit group (Table 2). Similarly, among those who were younger (age <65 years), FI-identified severe (HR: 1.99; 95% CI: 1.52-2.61) and moderate frailty (HR: 1.41; 95% CI: 1.14-1.76) remained significantly associated with a higher mortality probability, whereas FRAIL-identified mild and moderate-to-severe frailty did not (Table 2).

For secondary outcomes, discrepancies in outcome associations were observed between FRAIL scale and FI results. After adjustment, FRAIL-identified moderate-to-severe frailty (IRR: 1.2; 95% CI: 1.09-1.32) was independently associated with a higher probability of all-cause hospitalization than the robust group (Table 3). FI-identified severe (IRR: 1.38; 95% CI: 1.33-1.43), moderate (IRR: 1.20; 95% CI: 1.17-1.24), and mild frailty (IRR: 1.15; 95% CI: 1.12-1.18) were also associated with a significantly higher hospitalization probability than the fit group. However, FRAIL-identified moderate-to-severe frailty remained significantly associated with a higher probability of ICU admission (IRR: 4.19; 95% CI: 1.69-10.38), whereas FI-identified severe, moderate, and mild frailty was not (Table 3). FRAIL-identified moderate-to-severe (IRR: 1.46; 95% CI: 1.28-1.66) and mild frailty (IRR: 1.07; 95% CI: 1.03-1.12) correlated with an increased CVD-related hospitalization probability. Similar risk stratification effects were observed if we stratified patients according to FI severity categories (severe, moderate, and mild, IRR: 1.36, 1,24, and 1.19; 95% CI: 1.29-1.44, 1.18-1.29, and 1.14-1.24, respectively) (Table 3). Among older adults, there remains outcome correlation concordance with regard to all-cause hospitalization between FRAIL scale (for FRAIL-identified moderate-to-severe frailty, IRR: 1.18; 95% CI: 1.05-1.33) and FI (for FI-identified severe and moderate frailty, IRR: 1.25 and 1.11; 95% CI: 1.19-1.31 and 1.07-1.15, respectively) (Supplemental Table 2). Similar findings were observed regarding CVD-related hospitalization between the 2 instruments. Among older adults, FRAIL-identified moderate-to-severe frailty remained significantly associated with a higher probability of ICU admission (IRR: 3.57; 95% CI: 1.17-10.91), whereas FI-identified severe, moderate, and mild frailty was not (Supplemental Table 2). Among younger patients, the trend of results remained essentially similar (Supplemental Table 2). Sensitivity analyses were performed for the subgroup of patients with type 2 DM receiving antidiabetic drugs (Table 4). Similar findings were obtained regarding the outcome associations of the FRAIL scale and FI results.

**Table 3:** Risk of Secondary Outcomes Associated With 2 Frailty Assessment Results Among the Entire Cohort

	Number of Events	Total Population	Person-Years	Incidence Density^a^	Crude		Model 1^b^		Model 2^c^	
					IRR:	95% CI	IRR:	95% CI	IRR:	95% CI
All-cause hospitalization										
FRAIL scale										
0	39,636	25,061	158,781.9	249.6	1.00		1.00		1.00	
1	8,795	4,659	20,655.6	425.8	1.71	1.67-1.75^d^	0.96	0.93-0.99^d^	0.99	0.96-1.02
≧2	461	292	975.6	472.5	1.89	1.73-2.07^d^	1.05	0.96-1.16	1.20	1.09-1.32^d^
Per 1 score					1.62	1.59-1.65^d^	0.98	0.95-1.00	1.02	0.99-1.05
Frail index										
Fit	13,399	9,068	64,714.7	207.0	1.00		1.00		1.00	
Mild	10.116	6,128	39,609.9	255.4	1.23	1.20-1.27^d^	1.14	1.11-1.18^d^	1.15	1.12-1.18^d^
Moderate	13,840	8,282	47,071.4	294.0	1.42	1.39-1.45^d^	1.19	1.16-1.23^d^	1.20	1.17-1.24^d^
Severe	11,537	6,534	29,017.1	397.6	1.92	1.87-1.97^d^	1.34	1.29-1.38^d^	1.38	1.33-1.43^d^
Per 1 quartile					1.23	1.22-1.24^d^	1.09	1.08-1.11^d^	1.10	1.09-1.12^d^
ICU admission										
FRAIL scale										
0	137	25,061	158,781.9	0.9	1.00		1.00		1.00	
1	44	4,659	20,655.6	2.1	2.47	1.76-3.47^d^	1.30	0.83-2.05	1.39	0.88-2.18
≧2	6	292	975.6	6.1	7.13	3.15-16.14^d^	3.31	1.35-8.13^d^	4.19	1.69-10.38^d^
Per 1 score					2.49	1.90-3.26^d^	1.48	1.02-2.16^e^	1.61	1.10-2.35^e^
Frail index										
Fit	41	9,068	64,714.7	0.6	1.00		1.00		1.00	
Mild	37	6,128	39,609.9	0.9	1.47	0.95-2.30	1.23	0.77-1.96	1.24	0.78-1.99
Moderate	58	8,282	47,071.4	1.2	1.94	1.30-2.90^d^	1.31	0.83-2.08	1.32	0.83-2.10
Severe	51	6,534	29,017.1	1.8	2.77	1.84-4.18^d^	1.24	0.71-2.17	1.31	0.75-2.29
Per 1 quartile					1.40	1.23-1.59^d^	1.08	0.90-1.28	1.09	0.92-1.30
CVD hospitalization										
FRAIL scale										
0	17,038	25,061	158,7781.9	107.3	1.00		1.00		1.00	
1	4,676	4,659	260,655.6	226.4	2.11	2.04-2.18^d^	1.03	0.99-1.08	1.07	1.03-1.12^d^
≧2	252	292	975.6	258.3	2.41	2.13-2.73^d^	1.22	1.07-1.38^d^	1.46	1.28-1.66^d^
Per 1 score					1.94	1.88-1.99^d^	1.05	1.01-1.09^e^	1.11	1.06-1.15^d^
Frail index										
Fit	42,63	9,068	64,714.7	65.9	1.00		1.00		1.00	
Mild	4,486	6,128	39,609.9	113.3	1.72	1.65-1.79^d^	1.17	1.12-1.23^d^	1.19	1.14-1.24^d^
Moderate	6,883	8,282	47,071.4	146.2	2.22	2.14-2.31^d^	1.22	1.16-1.27^d^	1.24	1.18-1.29^d^
Severe	6,334	6,534	29,017.1	218.3	3.31	3.19-3.44^d^	1.29	1.22-1.36^d^	1.36	1.29-1.44^d^
Per 1 quartile					1.47	1.45-1.48^d^	1.08	1.06-1.10^d^	1.10	1.08-1.12^d^

CVD = cardiovascular disease; IRR = incidence rate ratio.

a Per 1,000 patient-years.

b Incorporating demographic and physical data, comorbidities, and medications.

c Incorporating model 1 variables and laboratory data.

d *P* < 0.01.

e *P* < 0.05.

**Table 4:** Sensitivity Analysis Including Only Patients With Diabetes Receiving Antidiabetic Drugs

	Number of Events	Total Population	Person-Years	Incidence Density^a^	Crude		Model 1^b^		Model 2^c^	
					HR	95% CI	HR	95% CI	HR	95% CI
Mortality										
FRAIL scale										
0	1,395	17,743	132,786.0	10.51	1.00		1.00		1.00	
1	410	3,065	16,804.6	24.40	2.53	2.26-2.82^d^	0.99	0.85-1.15	1.01	0.86-1.17
≧2	15	159	589.8	25.43	2.93	1.76-4.87^d^	0.95	0.57-1.61	1.03	0.61-1.74
Per 1 score					2.30	2.08-2.53^d^	0.98	0.85-1.13	1.00	0.87-1.15
Frail index										
Fit	413	6,588	54,572.8	7,57	1.00		1.00		1.00	
Mild	365	4,431	33,686.5	10.84	1.46	1.27-1.68^d^	1.19	1.02-1.38^e^	1.19	1.02-1.38^e^
Moderate	548	5,805	39,327.2	13.93	1.95	1.72-2.22^d^	1.29	1.12-1.50^d^	1.27	1.09-1.47^d^
Severe	494	4,143	22,593.8	21.86	3.28	2.87-3.74^d^	1.51	1.27-1.80^d^	1.49	1.25-1.78^d^
Per 1 quartile					1.47	1.41-1.53^d^	1.14	1.08-1.21^d^	1.13	1.07-1.20^d^

a Per 1,000 patient-years.

b Incorporating demographic and physical data, comorbidities, and medications.

c Incorporating model 1 variables and laboratory data.

d *P* < 0.01.

e *P* < 0.05.

## Discussion

In this study, we evaluated a large cohort of Asian patients with type 2 DM regarding the association between frailty assessment tools and multiple outcomes. We found that despite fair correlation of results, FRAIL-identified higher frailty severity was significantly associated with an increased probability of all-cause hospitalization, ICU admission, and CVD-related hospitalization. In contrast, FI-identified higher frailty severity was associated with the probability of mortality, all-cause hospitalization, and CVD-related hospitalization (Central Illustration). Our findings support the notion that the outcomes being evaluated in Asian DM patients should dictate the choice of frailty assessment tools.

**Central Illustration:**
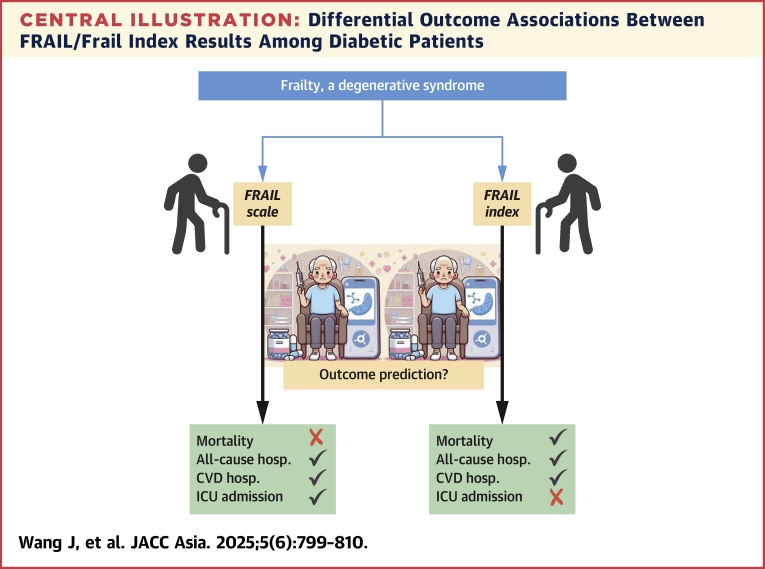
Differential Outcome Associations Between FRAIL/Frail Index Results Among Diabetic Patients Black checkmark signifies the presence of independent associations, whereas red X signifies absence. CVD = cardiovascular disease; hosp. = hospitalization; ICU = intensive care unit.

We discovered that the outcome influences differed according to the frailty assessment tools used (Tables 2 and 3), findings that were previously poorly characterized in Asian DM patients. Chode et al^20^ found that multiple frailty assessment tools (FRAIL, Cardiovascular Health Scale, Study of Osteoporotic Fractures scale, and Fried phenotype) identified frail DM patients, but they did not compare the degree of outcome correlation between each tool. In other populations, studies adopting approaches similar to ours exist. The FRAILTOOLS project showed that no single frailty assessment tool outperformed the others regarding outcome prediction in older adults^21^; FRAIL scale results independently predicted mortality but not hospitalization risk, whereas Fried phenotype predicted hospitalization risk but not fall. They concluded that the outcome predictability depended on frailty-assessment instrument types and the clinical context.^21^ However, these findings may not be applicable to those with DM. Therefore, our findings help fill the evidence gap by informing the choice of appropriate frailty assessment tools in DM patients, particularly in Asian populations.

The diagnosis of frailty further influences the management strategy for patients with DM. Studies already pinpoint frailty as an integral outcome modifier in clinical trials, and frail individuals may reap varying degrees of benefits from different kinds of interventions compared with nonfrail individuals.^22^ This is also the case for patients with DM. In the Dapagliflozin and Prevention of Adverse Outcomes in Heart Failure trial, patients with DM and frailty at baseline benefited more from the administered treatment than those without frailty.^23^ However, different frailty assessment tools yield divergent results, and whether results from one tool exhibit independent correlation with our outcomes of interest remains unclear, especially in the DM population. This issue is vital, because newer generations of antidiabetic medications constitute the main pillars of therapies (eg, sodium-glucose cotransporter-2 inhibitors and others), and there are concerns that these medications may alter frailty or sarcopenia severity.^24^ How we recognize frailty thus influences the selection of appropriate candidates. It would be prudent to better understand the relationship between different frailty assessment tools and the risk of different outcomes.

Notably, FI results and FRAIL scale results exhibited different outcome correlations (Tables 2 and 3). Several reasons may be responsible for these findings. First, patients with frailty diagnosed by different tools can have distinct features leading to prognoses diversification. A network meta-analysis reveals that existing frailty assessment tools exhibit different diagnostic accuracy.^25^ Others also concluded that frailty screening instruments differed in their sensitivity and specificity.^26^ In addition, eFIs constructed based on electronic health care records also manifest different sensitivity and specificity for frailty detection, as shown by Brack et al.^27^ It is plausible that the FRAIL scale, because of its comparatively lower sensitivity for identifying frailty, failed to recognize sufficient numbers of patients for satisfying the statistical efficiency required for mortality risk discrimination, whereas the FI did. Nonetheless, FRAIL-identified frail individuals might have a greater frailty severity that predisposes them to critical care requirements relatively to FI-identified ones. Anecdotal reports yielded similar findings.^28^ Second, the number of tool components may play a role. Our FI contains 38 items, but the FRAIL scale only assesses 5 items; the latter may be at a disadvantage when quantifying frailty severity. A population-based study showed that a 14-item FI exhibited superior mortality predictive efficacy compared with a 5-item one.^29^ Nonetheless, more studies are needed to validate our findings.

Our findings may have potentially important clinical implications. Individualizing treatment goals has been pivotal in devising care plans both for older adults and those with DM. Frailty, with its complex inter-relationship with other morbidities,^30^ has become essential in DM management. The development of frailty in patients with DM prominently worsens their outcomes. Moreover, the management of frailty also evolves stably over time.^31^ Therefore, the identification of frailty in these patients signifies that their glycemic control strategies may need fine tuning, care plans need to take into account frailty management, and their treatment preferences may need adjudication. Importantly, patients do not value each outcome equally and tend to emphasize certain issues over others. Some may emphasize survival at all cost, whereas others choose to avoid hospitalization or ICU care. It is thus imperative that physicians provide individualized recommendations for patients with DM, especially an appropriate frailty assessment tool, so as to prepare them for subsequent reformulation of management plans. This should incorporate the outcome influences valued by the patients most, be it mortality or not, because different tools yield results correlating with specific outcome types. Based on our findings, we should carefully select the frailty assessment tool in this population to facilitate our provision of holistic care.

### Study limitations

The topic of this study, the frailty tool-outcome relationship, is of clinical importance but has rarely been addressed in patients with DM. To answer this question, we evaluated a large cohort of patients with type 2 DM from a representative population and collected a wide range of potential confounders and different types of variables for adjustment. Nonetheless, several issues warrant consideration before interpreting these findings. First, we did not reference the Fried phenotype. However, the FRAIL scale results have been shown to correlate significantly with Fried phenotype results in Chinese patients.^32^ Our FI also exhibits fair validity and prognosis association in local population.^17^ Second, our results might not be applicable to patients with other comorbidities because we only included those with DM. Third, the treatment intensities assigned to patients with different frailty severities may differ, introducing occult influences on the association between frailty severities and patients’ outcomes. However, we found no significant associations between frailty severities (FRAIL scale or FI scores) and the probability of receiving hospice/palliative care among our patients with DM (Supplemental Table 3), largely excluding this possibility. Fourth, our patients were ethnically homogeneous, and extrapolating these findings to other countries may require careful adjudication.

## Conclusions

From a large cohort of patients with type 2 DM, we discovered that both FRAIL and FI identified a subgroup of patients with DM at significantly higher probability of all-cause and CVD-related hospitalization. However, FI failed to identify those having a higher probability of receiving critical care, whereas FRAIL was unable to identify those at an increased probability of mortality. Because care preferences, glycemic control strategies, and outcomes of patients with DM can be substantially altered by the presence of frailty, we should carefully select the frailty assessment tool when providing care for this population.

## Funding Support and Author Disclosures

This work was supported by National Taiwan University Hospital (no. 114-N0054), National Science and Technology Council, Taiwan (NSTC 112-2314-B-002-232-MY3), and Min Sheng General Hospital. The sponsors have no role in the study design, data collection, analysis, and result interpretation of this study. The authors have reported that they have no relationships relevant to the contents of this paper to disclose.
