# Supplementary material for: Frailty Assessment Tools Influence the Outcome Associations Among Patients With Diabetes: A Retrospective Cohort Study
Source: JACC Asia. 2025 Apr 22;5(6):799–810. doi: 10.1016/j.jacasi.2025.02.014 (PMC12287743; doi:10.1016/j.jacasi.2025.02.014)
Supplement: Supplemental Tables 1-3 [file mmc1.docx]

**Supplemental Table 1**. Clinical features of study cohort according to the frailty index results

|  | **Frailty index results** | | | | ***p-value*** |
| --- | --- | --- | --- | --- | --- |
|  | **Fit (n = 9,068)** | **Mild (n = 6,128)** | **Moderate (n = 8,282)** | **Severe (n = 6,534)** |  |
| *Demographic and physical data* |  |  |  |  |  |
| Age (years) | 59.8 ± 10.0 | 63.0 ± 10.2 | 65.4 ± 10.4 | 69.7 ± 10.9 | *<0.01* |
| Sex (Female %) | 4,097 (45.2) | 2,731 (44.6) | 3,713 (44.8) | 3,085 (47.2) | *<0.01* |
| BMI (IQR) | 24.8 (22.5, 27.3) | 25.2 (22.9, 28.0) | 25.2 (22.8, 27.8) | 25.0 (22.6, 27.7) | *<0.01* |
| *Comorbidity profile* |  |  |  |  |  |
| Hypertension (%) | 3,097 (34.2) | 4,215 (68.8) | 6,383 (77.1) | 5,571 (85.3) | *<0.01* |
| Hyperlipidemia (%) | 3,913 (43.2) | 2,998 (48.9) | 4,249 (51.3) | 3,554 (54.4) | *<0.01* |
| Atrial fibrillation (%) | 103 (1.1) | 289 (4.7) | 917 (11.1) | 1,426 (21.8) | *<0.01* |
| Acute coronary syndrome (%) | 756 (8.3) | 1,737 (28.4) | 2,895 (35.0) | 2,865 (43.9) | *<0.01* |
| Congestive heart failure (%) | 51 (0.6) | 164 (2.7) | 689 (8.3) | 1,153 (17.7) | *<0.01* |
| Cerebrovascular disease (%) | 22 (0.2) | 89 (1.5) | 462 (5.6) | 1,042 (16.0) | *<0.01* |
| Prior myocardial infarction (%) | 116 (1.3) | 201 (3.3) | 379 (4.6) | 383 (5.9) | *<0.01* |
| Peripheral vascular disease (%) | 110 (1.2) | 118 (1.9) | 233 (2.8) | 367 (5.6) | *<0.01* |
| Chronic kidney disease (%) | 402 (4.4) | 593 (9.7) | 1,345 (16.2) | 1,946 (29.8) | *<0.01* |
| Cancer (%) | 1,020 (11.3) | 638 (10.4) | 983 (11.9) | 1,012 (15.5) | *<0.01* |
| Chronic liver disease (%) | 1,192 (13.2) | 696 (11.4) | 964 (11.6) | 962 (14.7) | *<0.01* |
| COPD (%) | 38 (0.4) | 48 (0.8) | 124 (1.5) | () | *<0.01* |
| *Medication usage* |  |  |  |  |  |
| **Anti-hypertensives** |  |  |  |  |  |
| ACEI (%) | 546 (6.0) | 548 (8.9) | 670 (8.1) | 468 (7.2) | *<0.01* |
| ARB (%) | 2,503 (27.6) | 2,734 (44.6) | 4,032 (48.7) | 3,329 (51.0) | *<0.01* |
| β-blockers (%) | 1,370 (15.1) | 1,686 (27.5) | 2,352 (28.4) | 1,871 (28.6) | *<0.01* |
| CCB (%) | 1,765 (19.5) | 2,205 (36.0) | 3,322 (40.1) | 2,977 (45.6) | *<0.01* |
| Diuretics (%) | 1,143 (12.6) | 1,303 (21.3) | 2,285 (27.6) | 2,252 (34.5) | *<0.01* |
| α-blockers (%) | 287 (3.2) | 461 (7.5) | 875 (10.6) | 890 (13.6) | *<0.01* |
| **Anti-lipidemics (%)** | 3,662 (40.4) | 2,929 (47.8) | 3,986 (48.1) | 3,043 (46.6) | *<0.01* |
| **Anti-platelets (%)** | 1,700 (18.8) | 2,374 (38.7) | 3,831 (46.3) | 3,267 (50.0) | *<0.01* |
| **Anti-coagulants (%)** | 140 (1.5) | 135 (2.2) | 305 (3.7) | 479 (7.3) | *<0.01* |
| **Non-steroidal Anti-inflammatory drugs (%)** | 401 (4.4) | 543 (8.9) | 1,119 (13.5) | 1,478 (22.6) | *<0.01* |
| **Anti-diabetic drugs** |  |  |  |  | *<0.01* |
| OAD monotherapy (%) | 1,880 (20.7) | 1,417 (23.1) | 1,986 (24.0) | 1,719 (26.3) |  |
| OAD combination therapy (%) | 4,131 (45.6) | 2,587 (42.2) | 3,258 (39.3) | 1,917 (29.3) |  |
| Insulin (%) | 577 (6.4) | 427 (7.0) | 561 (6.8) | 507 (7.8) |  |
| *Laboratory data* |  |  |  |  |  |
| Fasting glucose (mg/dL) | 138.1 ± 47.3 | 134.7 ± 52.2 | 129.7 ± 43.8 | 124.5 ± 48.1 | *<0.01* |
| Glycated hemoglobin (%) | 7.28 ± 1.50 | 7.16 ± 1.42 | 6.99 ± 1.34 | 6.76 ± 1.25 | *<0.01* |
| Total cholesterol (mg/dL) | 186.44 ± 41.35 | 183.12 ± 40.65 | 180.76 ± 41.78 | 177.65 ± 41.29 | *<0.01* |
| Triglyceride (mg/dL) | 157.4 ± 123.6 | 161.0 ± 116.6 | 159.5 ± 132.1 | 155.4 ± 107.0 | *0.21* |
| HDL cholesterol (mg/dL) | 43.7 ± 11.4 | 43.0 ± 11.1 | 42.9 ± 11.5 | 43.1 ± 12.2 | *0.16* |
| LDL cholesterol (mg/dL) | 107.45 ± 34.50 | 104.13 ± 33.60 | 102.91 ± 35.21 | 100.10 ± 32.58 | *<0.01* |
| Creatinine (mg/dL) | 1.0 ± 0.6 | 1.1 ± 1.0 | 1.3 ± 1.4 | 1.5 ± 1.7 | *<0.01* |
| eGFR (mL/min/1.73m^2^) | 78.4 ± 22.0 | 74.5 ± 24.4 | 70.7 ± 26.4 | 67.8 ± 29.9 | *<0.01* |
| AST (U/L) | 29.36 ± 37.12 | 28.49 ± 28.25 | 29.71 ± 91.89 | 28.61 ± 33.22 | *0.61* |
| ALT (U/L) | 32.4 ± 35.7 | 30.6 ± 29.7 | 28.6 ± 29.3 | 26.2 ± 27.6 | *<0.01* |
| *Frailty assessment results* |  |  |  |  |  |
| Frail index | 0.03 (0.00, 0.03) | 0.05 (0.05, 0.05) | 0.08 (0.08, 0.11) | 0.16 (0.13, 0.21) | *<0.01* |
| FRAIL scale | 0.0 (0.0, 0.0) | 0.0 (0.0, 0.0) | 0.0 (0.0, 0.0) | 0.0 (0.0, 0.0) | *<0.01* |

*ACEI, angiotensin-converting enzyme inhibitor; ARB, angiotensin receptor blocker; ALT, alanine transaminase; AST, aspartate transaminase; BMI, body mass index; CCB, calcium channel blocker; COPD, chronic obstructive pulmonary disease; eGFR, estimated glomerular filtration rate; HDL, high density lipoprotein; IQR, interquartile range; LDL, low density lipoprotein; OAD, oral antidiabetic drug*

**Supplemental Table 2.** Risk of secondary outcomes associated with two frailty assessment results among patients in different age strata

| **Variables** | **Number of events** | **Total population** | **Person-years** | **Incidence density*** | **Model^&^** | |
| --- | --- | --- | --- | --- | --- | --- |
|  |  |  |  |  | **IRR** | **95% CI** |
| ***Age ≥ 65*** | | | | | | |
| ***All-cause hospitalization*** | |  |  |  |  |  |
| *FRAIL scale* | |  |  |  |  |  |
| 0 | 20,055 | 10,814 | 67,240.2 | 298.3 | 1.00 |  |
| 1 | 5,629 | 2,827 | 11,911.7 | 472.6 | 1.05 | 1.01-1.09^†^ |
| ≧2 | 298 | 187 | 577.9 | 515.7 | 1.18 | 1.05-1.33^††^ |
| Per 1 score |  |  |  |  | 1.06 | 1.02-1.10^††^ |
| *Frail index* | |  |  |  |  |  |
| Fit | 4,969 | 2,676 | 18,988.8 | 261.5 | 1.00 |  |
| Mild | 5,084 | 2,564 | 16,928.5 | 300.3 | 1.08 | 1.04-1.13^††^ |
| Moderate | 7,994 | 4,267 | 24,800.5 | 322.3 | 1.11 | 1.07-1.15^††^ |
| Severe | 7,935 | 4,321 | 19,002.0 | 417.6 | 1.25 | 1.19-1.31^††^ |
| Per 1 quartile |  |  |  |  | 1.07 | 1.06-1.09^††^ |
| ***ICU admission*** |  |  |  |  |  |  |
| *FRAIL scale* | |  |  |  |  |  |
| 0 | 89 | 10,814 | 67,240.2 | 1.3 | 1.00 |  |
| 1 | 30 | 2,827 | 11,911.7 | 2.5 | 1.08 | 0.62-1.89 |
| ≧2 | 4 | 187 | 577.9 | 6.9 | 3.57 | 1.17-10.91^†^ |
| Per 1 score |  |  |  |  | 1.33 | 0.83-2.15 |
| *Frail index* | |  |  |  |  |  |
| Fit | 26 | 2,676 | 18,998.8 | 1.4 | 1.00 |  |
| Mild | 26 | 2,564 | 16,928.5 | 1.5 | 0.88 | 0.50-1.56 |
| Moderate | 35 | 4,267 | 24,800.5 | 1.4 | 0.66 | 0.37-1.17 |
| Severe | 36 | 4,321 | 19,002.0 | 1.9 | 0.61 | 0.31-1.21 |
| Per 1 quartile |  |  |  |  | 0.84 | 0.67-1.04 |
| ***CVD hospitalization*** | |  |  |  |  |  |
| *FRAIL scale* | |  |  |  |  |  |
| 0 | 9,844 | 10,814 | 67,240.2 | 146.4 | 1.00 |  |
| 1 | 3,215 | 2,827 | 11,911.7 | 269.9 | 1.10 | 1.05-1.17^††^ |
| ≧2 | 183 | 187 | 577.9 | 316.7 | 1.40 | 1.20-1.63^††^ |
| Per 1 score |  |  |  |  | 1.12 | 1.07-1.18^††^ |
| *Frail index* | |  |  |  |  |  |
| Fit | 1,894 | 2,676 | 18,988.8 | 99.7 | 1.00 |  |
| Mild | 2,530 | 2,564 | 16,928.5 | 149.5 | 1.13 | 1.06-1.20^††^ |
| Moderate | 4,202 | 4,267 | 24,800.5 | 169.4 | 1.12 | 1.06-1.19^††^ |
| Severe | 4,616 | 4,321 | 19,002.0 | 242.9 | 1.23 | 1.15-1.32^††^ |
| Per 1 quartile |  |  |  |  | 1.06 | 1.04-1.08^††^ |
| ***Age < 65*** | | | | | | |
| ***All-cause hospitalization*** | |  |  |  |  |  |
| *FRAIL scale* | |  |  |  |  |  |
| 0 | 19,581 | 14,247 | 91,541.7 | 213.9 | 1.00 |  |
| 1 | 3,166 | 1,832 | 8,744.0 | 362.1 | 0.96 | 0.92-1.01 |
| ≧2 | 163 | 105 | 397.7 | 409.8 | 1.23 | 1.05-1.44^†^ |
| Per 1 score |  |  |  |  | 0.99 | 0.95-1.04 |
| *Frail index* | |  |  |  |  |  |
| Fit | 8,430 | 6,392 | 45,715.9 | 184.4 | 1.00 |  |
| Mild | 5,032 | 3,564 | 22,681.4 | 221.9 | 1.20 | 1.15-1.24^††^ |
| Moderate | 5,846 | 4,015 | 22,271.0 | 262.5 | 1.29 | 1.24-1.34^††^ |
| Severe | 3,602 | 2,213 | 10,015.1 | 359.7 | 1.51 | 1.43-1.59^††^ |
| Per 1 quartile |  |  |  |  | 1.14 | 1.12-1.16^††^ |
| ***ICU admission*** |  |  |  |  |  |  |
| *FRAIL scale* | |  |  |  |  |  |
| 0 | 48 | 14,247 | 91,541.7 | 0.5 | 1.00 |  |
| 1 | 14 | 1,832 | 8,744.0 | 1.6 | 2.22 | 1.02-4.84^†^ |
| ≧2 | 2 | 105 | 397.7 | 5.0 | 5.89 | 1.21-28.75^†^ |
| Per 1 score |  |  |  |  | 2.25 | 1.20-4.21^†^ |
| *Frail index* | |  |  |  |  |  |
| Fit | 15 | 6,392 | 45,715.9 | 0.3 | 1.00 |  |
| Mild | 11 | 3,564 | 22,681.4 | 0.5 | 1.96 | 0.87-4.41 |
| Moderate | 23 | 4,015 | 22,271.0 | 1.0 | 3.95 | 1.87-8.32^††^ |
| Severe | 15 | 2,213 | 10,015.1 | 1.5 | 5.26 | 2.06-13.40^††^ |
| Per 1 quartile |  |  |  |  | 1.79 | 1.34-2.39^††^ |
| ***CVD hospitalization*** | |  |  |  |  |  |
| *FRAIL scale* | |  |  |  |  |  |
| 0 | 7,194 | 14,247 | 91,541.7 | 78.6 | 1.00 |  |
| 1 | 1,461 | 1,832 | 8,744.0 | 167.1 | 1.05 | 0.97-1.13 |
| ≧2 | 69 | 105 | 397.7 | 173.5 | 1.54 | 1.21-1.97^††^ |
| Per 1 score |  |  |  |  | 1.09 | 1.02-1.17^†^ |
| *Frail index* | |  |  |  |  |  |
| Fit | 2,369 | 6,392 | 45,715.9 | 51.8 | 1.00 |  |
| Mild | 1,956 | 3,564 | 22,681.4 | 86.2 | 1.20 | 1.12-1.28^††^ |
| Moderate | 2,681 | 4,015 | 22,271.0 | 120.4 | 1.36 | 1.27-1.45^††^ |
| Severe | 1,718 | 2,213 | 10,015.1 | 171.5 | 1.52 | 1.40-1.66^††^ |
| Per 1 quartile |  |  |  |  | 1.15 | 1.12-1.18^††^ |

*CI, confidence interval; CVD, cardiovascular disease; IRR, incidence rate ratio*

^*^ per 1000 patient-year

^&^ Incorporating demographic and physical data, comorbidities, medications, and laboratory data

^†^*p < 0.05*

^††^*p < 0.01*

**Supplemental Table 3.** Probability of receiving hospice/palliative care according to frailty status

| **Variables** | **Number of events** | **Total population** | **Person-years** | **Incidence density*** | **Model ^&^** | |
| --- | --- | --- | --- | --- | --- | --- |
|  |  |  |  |  | **IRR** | **95% CI** |
| *FRAIL scale* | |  |  |  |  |  |
| 0 | 248 | 25,061 | 158,781.9 | 1.6 | 1.00 |  |
| 1 | 69 | 4,659 | 20,655.6 | 3.3 | 1.06 | 0.75-1.50 |
| ≧2 | 6 | 292 | 975.6 | 6.1 | 1.65 | 0.69-3.96 |
| Per 1 score |  |  |  |  | 1.18 | 0.87-1.60 |
| *Frail index* | |  |  |  |  |  |
| Fit | 107 | 9,068 | 64,714.7 | 1.7 | 1.00 |  |
| Mild | 52 | 6,128 | 39,609.9 | 1.3 | 0.78 | 0.55-1.11 |
| Moderate | 76 | 8,282 | 47,071.4 | 1.6 | 0.86 | 0.62-1.20 |
| Severe | 88 | 6,534 | 29,017.1 | 3.0 | 1.83 | 0.91-1.93 |
| Per 1 quartile |  |  |  |  | 1.20 | 0.95-1.22 |

*CI, confidence interval; IRR, incidence rate ratio*

^*^ per 1000 patient-year

^&^ Incorporating demographic and physical data, comorbidities, medications, and laboratory data
